# Poly (ADP-Ribose) Polymerase-1 (PARP-1) Inhibitors in Diabetic Retinopathy: An Attractive but Elusive Choice for Drug Development

**DOI:** 10.3390/pharmaceutics16101320

**Published:** 2024-10-11

**Authors:** Etelka Pöstyéni, Róbert Gábriel, Andrea Kovács-Valasek

**Affiliations:** 1 Department of Experimental Zoology and Neurobiology, University of Pécs, Ifjúság útja 6, 7624 Pécs, Hungary; etelka91@gamma.ttk.pte.hu (E.P.); valasek@gamma.ttk.pte.hu (A.K.-V.); 2 János Szentágothai Research Centre, Ifjúság útja 20, 7624 Pécs, Hungary

**Keywords:** PARP-1, retina, diabetic retinopathy, therapy

## Abstract

Owing to its promiscuous roles, poly (ADP-ribose) polymerase-1 (PARP-1) is involved in various neurological disorders including several retinal pathologies. Diabetic retinopathy (DR) is the most common microvascular complication of diabetes mellitus affecting the retina. In the present review, we highlight the importance of PARP-1 participation in pathophysiology of DR and discuss promising potential inhibitors for treatment. A high glucose level enhances PARP-1 expression; PARP inhibitors have gained attention due to their potential therapeutic effects in DR. They target different checkpoints (blocking nuclear transcription factor (NF-κB) activation; oxidative stress protection, influence on vascular endothelial growth factor (VEGF) expression, impacting neovascularization). Nowadays, there are several improved clinical PARP-1 inhibitors with different allosteric effects. Combining PARP-1 inhibitors with other compounds is another promising option in DR treatments. Besides pharmacological inhibition, genetic disruption of the PARP-1 gene is another approach in PARP-1-initiated therapies. In terms of future treatments, the limitations of single-target approaches shift the focus onto combined therapies. We emphasize the importance of multi-targeted therapies, which could be effective not only in DR, but also in other ischemic conditions.

## 1. Introduction

The PARP (poly (ADP-ribose) polymerase) superfamily contains more than 17 different members, which have diverse distribution, expression patterns and pleiotropic roles in various physiological cellular processes, such as DNA repair, gene expression regulation and cell death. These proteins are evolutionary-conserved and essential both in abiotic (e.g., oxidative stress) and biotic stress (e.g., pathogen infection) responses. In normal conditions, PARP-KO mice models do not show significant alterations in their phenotypes, until they are under the influence of different stress factors [1,2].

PARP-1 is the most prevalent and abundant DNA-dependent nuclear enzyme of this family, its activity is responsible for more than 90% of the generated poly (ADP-ribose) (PARs). It contains three different domains: an amino-terminal DNA-binding domain, a central automodification domain and a highly conserved C-terminal catalytic domain. Additionally, PARP-1 possesses other functional regions, such as the ankyrin repeat region of anchor polymerase and the poly (ADP-ribose) polymer binding region [3,4,5]. Its biological significance has been proven in several subcellular mechanisms such as in gene expression regulation (RNA regulation, transcriptional regulation, chromatin modification) [1], in DNA damage responses (DNA repair mechanisms), in tumor development and progression, in cell death (parthanatos, necroptosis and autophagy), in lipid metabolism and also in inflammatory processes [6,7]. PARP-1 works as “guardian of the genome” because it is activated after genotoxic stress, which causes structural changes in the genome. This is the main reason why PARP-1 defective cells are more sensitive to DNA damage [8].

PARP-1 detects and responds to single-strand DNA breaks by initiating DNA repair processes via catalyzing the ribosylation of poly ADP in the so-called PARylation reaction. It cleaves nicotinamide adenine dinucleotide (NAD^+^) and synthesizes negatively charged poly (ADP-ribose) (PAR) polymers on various target proteins, such as on histones, DNA repair enzymes and transcription factors, which are later degraded via poly (ADP-ribose) glycohydrolase. Its activity leads to the accumulation of PARs (50–200 PAR polymer) and the depletion of its substrate NAD^+^ [9,10,11]. The toxicity of elevated PARP, combined with the depletion of NAD^+^ and ATP, results in various cellular dysfunctions and cell death. Parthanatos, a PAR-dependent, capase-independent cell death mechanism, is important in neurons. When PAR polymers enter the cytoplasm, they bind to apoptosis-inducing factor mitochondrial oxidoreductase, which then associates with DNase, resulting in DNA fragmentation [12]. PARylation can modify RNA-binding proteins and influence RNA metabolism.

PARP-1, identified also as a downstream effector of reactive oxygen species (ROS), plays a critical role in cellular metabolism [1,13]. Its extensive activation depletes NAD^+^. However, restoring the NAD^+^ reservoir necessitates ATP consumption. Consequently, glycolysis and mitochondrial function are inhibited, ultimately leading to cellular dysfunction, apoptosis or necrosis [14].

PARP-1 serves as a downstream effector in both oxidative stress and nitrosative stress pathways, contributing to endothelial cell proliferation under hypoxic conditions. Effective antioxidants can mitigate ROS activity, enhance NAD^+^ levels, activate the retinal sirtuin-1 (SIRT-1) signaling pathway and safeguard the retina against oxidative damage induced by high glucose [15]. Interestingly, recent studies suggest bidirectional interactions between oxidative/nitrosative stress and PARP-1. Notably, in streptozotocin-induced diabetic mice, PARP-1 expression increased even in the absence of DNA single-strand damage in dorsal nerve roots [16]. This implies distinct mechanisms at play in diabetes or high-glucose states, differing from PARP-1 activation mechanisms during oxygen and nitrogen stress. The activation of PARP-1 disrupts mitochondrial membrane function, elevates ROS levels and impairs antioxidant enzymes like manganese superoxide dismutase, contributing to oxidative stress-related retinopathy [17,18].

PARP-1 does not only influence immune cell maturation and function but also modulates inflammatory responses. Furthermore, it promotes the activation of inflammatory factors and affects the expression of chemokines, cytokines, adhesion factors and mediators such as matrix metalloproteinases (MMP-2, MMP-3, MMP-9) [19,20,21,22], cyclooxygenase-2 and inducible nitric oxide synthase. Under inflammatory conditions, PARP-1 activation enhances oxidative stress responses and amplifies inflammation [23,24]. The activation of the AKT signaling pathway promotes cell survival, proliferation and migration. Studies reveal that hyperoxia induces peroxynitrite synthesis, leading to endothelial cell apoptosis, the upregulation of PARP-1 expression and the subsequent inhibition of the AKT pathway, resulting in vascular occlusion [25,26]. Under hyperglycemic conditions, Notch1 signaling protects cells from PARP-1- and nuclear transcription factor (NF-κB)-induced apoptosis by activating the AKT pathway [27,28,29]. NF-κB, a central regulator of inflammation, directly interacts with PARP-1 upon activation. This interaction leads to the increased expression of inflammatory factors, including tumor necrosis factor α (TNFα), interleukin 6 and intercellular adhesion factor 1 (ICAM-1) [23,30]. Notably, in a diabetic rat model, PARP-1 was associated with high mobility group box 1, an inflammatory factor that up-regulates diabetes-induced retinal cell apoptosis markers [31].

Owing to its multivarious roles in cell death and energy metabolism, PARP-1 is involved in various neurological disorders including retinal pathologies. The sight-threatening eye disorders (retinitis pigmentosa, age-related macular degeneration, glaucoma, etc.) are heterogenous and their treatments are usually unresolved. Due to diverse functions of PARP-1 in biological processes, it may offer a wide variety of molecular target points for possible treatments in these cases at a cellular level. The pharmacological inhibition or genetic disruption of PARP-1 has several promising effects on oxidative stress-caused abnormalities, inflammatory processes and ischemia.

## 2. Retinal Diseases and Poly (ADP-Ribose) Polymerase-1 (PARP-1)

**Figure 1:**
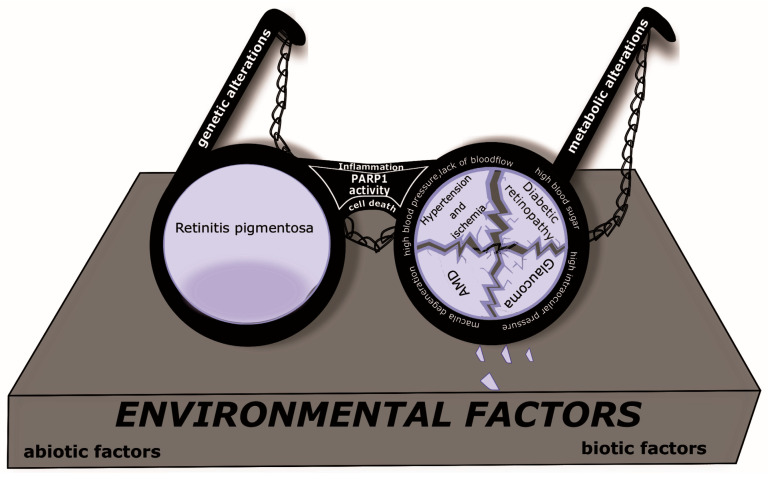
Retinal disorders through poly (ADP-ribose) polymerase-1’ (PARP-1) spectacles.

Retinal disorders can be divided into two main groups: genetic (e.g., retinitis pigmentosa) and metabolic (e.g., glaucoma, diabetic retinopathy and age-related macular degeneration). Inflammation and cell death are common features of retinal cell degeneration with both mentioned origins (Figure 1). Due to diversified roles of poly (ADP-ribose) polymerase-1 (PARP-1) in these molecular processes, its modification offers a wide variety of molecular target points for possible treatment options at a cellular level. Oxidative and nitrosative stress are strong risk factors in these pathologies and these are the main initiators of DNA damage, which initiate PARP-1 action. The behavior of PARP-1 depends on the severity of DNA damage: when it is mild it promotes cell survival, while, in the case of severe DNA damage, it facilitates cell death [32,33]. The inhibition of PARP-1 expression may also be an important target point in pharmacological research and shows promising results in the treatment of different retinal diseases. PARP-1 inhibitors are classified into three different types based on their exerted allosteric retention effects (allosteric pro-retention, non-allosteric and allosteric pro-release) [29,34]. Based on their main effect, PARP inhibitors also can be distinguished by catalytic inhibition and PARP-1 trapping [35]. Nowadays, there are several improved clinical PARP-1 inhibitors that are used in cancer treatments, such as olaparib, rucaparib and talazoparib. The combination of PARP inhibitors with other neuroprotective components may also be effective.

In addition to pharmacological inhibition, the genetic disruption of the PARP-1 gene is another approach in retinal research. PARP-1 knockout mice, although phenotypically normal, are protected from necrotic cell death and exhibit neurodevelopmental deficits [36,37].

## 3. Diabetic Retinopathy

Diabetic retinopathy (DR) is a tissue-specific neurovascular complication of diabetes mellitus and stands as a leading cause of vision impairment and blindness [38,39,40,41]. While the prevalence of DR varies among different types of diabetes, the global estimates indicate that approximately 22.27% to 27% of individuals with diabetes (both type I and type II) are affected [42]. In the Wisconsin Epidemiologic Study of Diabetic Retinopathy, also known as WESDR, researchers observed that among individuals with insulin-dependent diabetes (type I) diagnosed before age 30, the cumulative incidence of diabetic retinopathy over a 4-year period reached 59.0% [43]. Type II diabetes arises from a combination of risk factors, including age, heredity, smoking, obesity, excessive alcohol consumption, an unhealthy diet and a lack of physical inactivity. These factors disrupt glucose regulation and contribute to Type II diabetes development. Individuals diagnosed with Type II diabetes are at risk of organ damage, which falls into two primary categories: microvascular complications (such as nephropathy, retinopathy and neuropathy) and macrovascular complications (including stroke, cardiovascular problems and peripheral arterial diseases). Effective lifestyle interventions—such as maintaining regular physical activity, managing weight, adjusting dietary habits and quitting smoking—are essential for preventing Type II diabetes-related complications [44]. In addition to diabetes duration, or neglected glucose control, other risk factors contribute to DR. These include high blood pressure levels, hyperlipidemia, renal disease, overweight and physical inactivity. Furthermore, nearly 80% of individuals diagnosed with type II diabetes face the risk of developing retinopathy in ten years [45]. Specifically, in at least one eye, retinopathy occurs in 17.3% of patients, while bilateral DR affects 54.5% [46,47]. Additionally, the risk of DR increases for each year of both type I and type II diabetes duration [47].

The development and progression of DR results from various factors, including prolonged diabetes duration, inadequate blood glucose control and elevated blood pressure. Hyperglycemia contributes to the formation of microangiopathy, characterized by microaneurysms, hemorrhages and thickening of the basement membrane [48]. The grading of DR depends on whether the abnormal new blood vessels originate from the retina. Various stages can be distinguished. The less critical stage is called non-proliferative DR, which can be further subdivided into three phases. The mild phase is represented by microaneurysms only. The moderate phase is characterized by additional microaneurysm signs, along with findings such as cotton wool spots or dot/blot hemorrhages. The severe phase is defined by more than 20 intraretinal hemorrhages in each of the four quadrants, definite venous beading in two or more quadrants and prominent intraretinal microvascular abnormalities in one or more quadrant. The most serious stage, known as proliferative DR, is characterized by neovascularization and hemorrhage in the vitreous or preretinal space [39,49,50]. While these classifications have proven valuable for evaluating treatment effectiveness in research and providing general treatment guidelines, it is essential to recognize that each patient with DR presents a distinct combination of findings, symptoms and progression rates. As a result, a personalized treatment approach is necessary to protect vision.

Current treatments for DR emphasize prevention. This involves maintaining a healthy lifestyle through physical activity, proper diet and essential nutrients. Regulating blood sugar levels and managing psychosocial and oxidative stress are crucial for reducing the long-term mortality rate associated with diabetes [51]. In the context of preventing vascular complications, therapeutic options include the usage of natural and synthetic agents to target key elements (cyclooxygenase [52], nuclear transcription factor (NF-κB) [53], poly (ADP-ribose) polymerase (PARP) [54], tumor necrosis factor α (TNFα) [55]) in the diabetic-induced inflammatory response. Recently, multiple clinical studies support the management of intravitreal anti-VEGF (vascular endothelial growth factor) injections (such as bevacizumab) to decrease retinal neovascularization in patients suffering from DR [56,57,58]. In the severe phase of proliferative DR, laser photocoagulation techniques—such as peripheral retinal laser photocoagulation, focal macular laser photocoagulation and grid photocoagulation—are reliable for treating the condition. These approaches specifically target newly formed and abnormal vessels that leak [59].

To gain deeper insight into the complexities of DR, researchers have developed various animal models. These models play a crucial role in exploring the causes, progression and potential treatments of DR. However, no single animal model fully encompasses the entire range of vascular and neural complications observed at both early and late stages. Induced models involve surgical removal of the pancreas, drug administration (such as alloxan, streptozotocin, dithizone or ferric nitrilotriacetate for type I DR; and gold thioglucose for type II DR), high-galactose diets and laser or chemical damage. Genetic models are created via selective breeding and gene editing. Among the species employed—mice, rats, cats, dogs, pigs and nonhuman primates—mouse and rat models are the most common in research due to their compact size, brief lifespan and rapid reproductive rates. Interestingly, keeshond dog models exhibit DR phenotypes closest to those in humans [60], while nonhuman primates show relative resistance to induced DR [61]. Pigs and zebrafish are preferred for their eye structure similarity to humans, easily visualized vascular structures, short lifespans and large breeding capacities.

After undergoing pancreas removal surgery, adult cats exhibit hyperglycemia within three weeks. When alloxan is administered alongside a pancreatectomy, this process is accelerated, reducing the onset time to just one week. Furthermore, the thickening of the basement capillary membrane can manifest approximately three months after hyperglycemia begins. Over the later five to nine years, DR symptoms, including microaneurysms, intraretinal hemorrhages, capillary nonperfusion and neovascularization, gradually occur [62,63,64]. Research involving monkeys revealed that pancreatectomy, performed at varying ages between six and 15 years, resulted in insulin dependence and subsequent hyperglycemia. These monkeys exhibited BRB leakage within just one year of hyperglycemia onset. Interestingly, even after a decade postinduction, proliferative retinopathy did not emerge in these resilient primates [65].

Both alloxan and streptozotocin (STZ) are commonly used to induce experimental diabetes in animal models. They exert their diabetic properties by selectively damaging pancreatic β cells in the islets of Langerhans. Dithizone selectively binds to pancreatic β cells, staining them red and can cause β cell damage. The C57/Bl6 or FOT_FB mouse strain, following alloxan induction, exhibits rapid pericyte ghost formation and retinal ganglion cell loss within just 7 days. By day 21, microaneurysms emerge alongside increased acellular capillaries. At 3 months of age, microglial changes become evident, characterized by thicker cell bodies and shorter dendrites [66,67]. Alloxan administration in rats also promptly leads to hyperglycemia and diabetes within a week. Neovascularization occurs between 2 and 9 months postinduction, while pericyte ghosts, acellular capillaries and a thickened capillary basement membrane occur by 15 months postinduction. Additionally, this model displays blood–retinal barrier breakdown, the expansion of Müller glia and endothelial swelling [68,69]. Streptozotocin is now the preferred choice over alloxan for inducing diabetes, as it more accurately mimics the diabetic disease state.

Understanding the pathophysiological mechanisms of DR is crucial for effective management and prevention. Numerous studies have demonstrated that the diabetic state, characterized by hyperglycemia (defined as a random plasma glucose ≥ 11.1 mmol/L (200 mg/dL) or a fasting plasma glucose ≥ 7.0 mmol/L (126 mg/dL), involves multiple metabolic pathways. These include the polyol pathway, the accumulation of advanced glycation end products, the activation of the protein kinase C pathway, alterations in the hexosamine pathway and, ultimately, the activation of PARP. This activation is driven by the aforementioned pathways, which leads to the increased production of reactive oxygen species (ROS) [39,70].

### Poly (ADP-Ribose) Polymerase-1 (PARP-1) Inhibitors in Diabetic Retinopathy (DR)

Retinal cell death can occur in all neuronal layers, leading to early-stage vision dysfunction. The role of poly (ADP-ribose) polymerase-1 (PARP-1) in diabetic complications has been confirmed by various studies. For example, PARP-1 knockout mice did not develop diabetes when induced by the beta-cell toxin streptozotocin [71]. PARP-1 activation is present in neuropathy and vascular alterations of diabetic retinopathy (DR). A high glucose level enhances PARP-1 expression, which mediates glucose-induced vascular complications and inflammation [72]. With enhanced oxidative stress, DNA strands break, inducing PAPR-1 activation during the cellular pathology of DR. The PARP-1 mRNA level and the PARP expression increased in the inner nuclear layer (INL) and ganglion cell layer (GCL) of the diabetic rats’ retinas. [73]. In addition, He et al. (2015) conducted a comprehensive gene set enrichment analysis to identify pathways and transcription factors associated with DR. This study highlighted the significant role of PARP family members in DR [74].

As PARP-1 activation rises, it depletes NAD^+^ and ATP, which induces necrosis. Necrotic cells then release inflammatory mediators, making them defenseless against oxidative damage. The administration of antioxidants was protective against oxidative damage in the diabetic retina; moreover, it has been proved that the inhibition of PARP-1 can mitigate antioxidant capacity reduction in that condition [75,76,77]. Moreover, polyphenol-enriched cocoa treatment decreased PARP-1 activity and improved sirtuin-1 (SIRT-1) activity in animal models for hypertension and diabetes (SHR-STZ rats) [15]. After nicotinamide treatment, cleaved PARP-1 expression was decreased in the diabetic rats [78].

**Figure 2:**
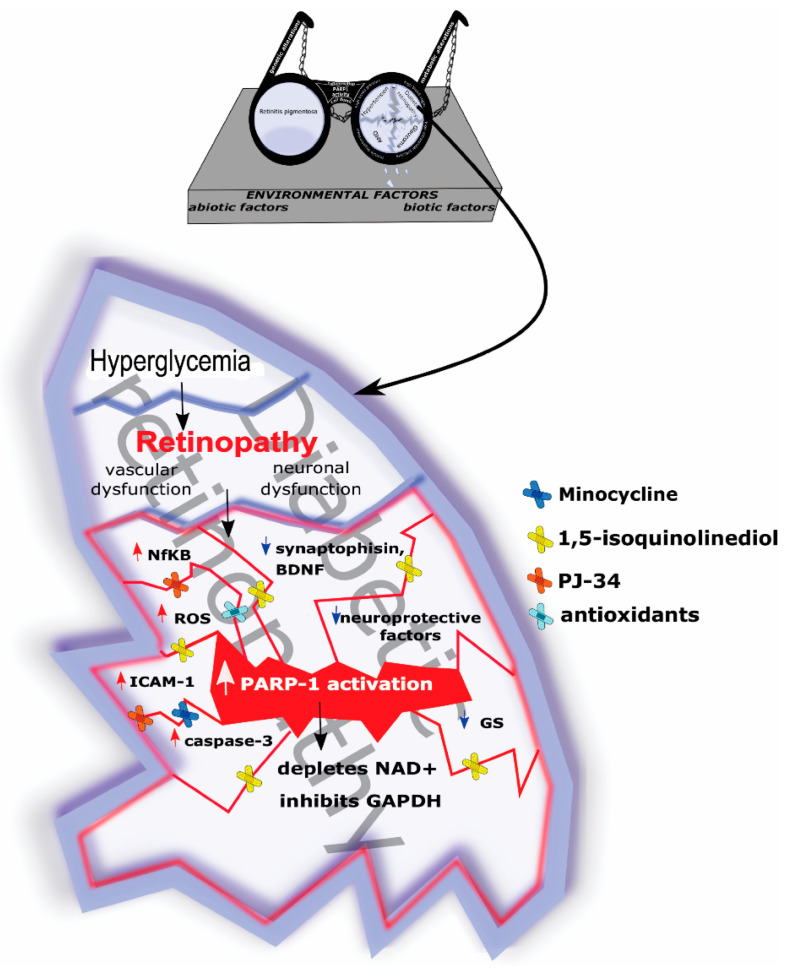
Effect of poly (ADP-ribose) polymerase (PARP) inhibitors in diabetic retinopathy (DR). Black arrows represent the principal events in the pathophysiology of DR, red arrows illustrate the rise of the substances, blue arrows indicate the decline of substances in cellular level.

PARP-1 immunoreactivity is upregulated in diabetic rat retinas, where PARP-1 inhibitors, including nicotinamide and 3-aminobenzamide, provide retinoprotective benefits by attenuating reactive gliosis and improving the angiogenic status. These inhibitors work by down-regulating PARP-1 and poly (ADP-ribose) (PARs), which in turn reduces damage to macroglia and prevents the formation of cleavage-derived products from PARP-1 and glial fibrillary acidic protein in the retina. Moreover, diabetic retinas often show decreased levels of angiostatin, a crucial regulator of angiogenesis. PARP-1 inhibitors help to restore angiostatin levels, thereby counteracting the abnormal pro-angiogenic state seen in diabetic retinopathy [79]. As a consequence of increased oxidative stress, the levels of brain-derived neurotrophic factor (BDNF), synaptophysin and glutamine synthetase (GS) were decreased in diabetic retinas. PARP inhibitors prevent the increase in NFκB signaling and promote BDNF and GS expression in diabetic rat retinas. The elevated level of caspase-3 and ERK-1/2 phosphorylation also were attenuated by the PARP-1 inhibitor, 1,5-isoquinolinediol [80]. Minocycline treatment could attenuate abnormal PARP-1 activation in DR. Furthermore, following 2 weeks of minocycline treatment, the apoptotic index in GCL and elevated caspase-3 level were suppressed; the electroretinogram (ERG) abnormalities were normalized compared to DR retinas [73]. Endothelial cell death, nuclear transcription factor (NF-κB) activation and intercellular adhesion factor 1 (ICAM-1) upregulation were suppressed by the PARP-1 inhibitor PJ-34 in diabetic rat retinas (Figure 2).

## 4. Poly (ADP-Ribose) Polymerase (PARP) Inhibitors in Combinations

Poly (ADP-ribose) polymerase-1 (PARP-1) inhibitors demonstrate a concentration-dependent response, where higher concentrations result in significant cytotoxic effects and cell death. Due to the risk, PARP-1 inhibitors should be used with caution when administered alone [12]. However, combinations of PARP inhibitors with other therapeutic agents are promising treatment strategies. The optimization of combinate therapies is a new approach in retinal disease research, although its usefulness is already well known in cancer treatment. The combination of PARP inhibitors (olaparib, veliparib, rucaparib, etc.) with chemotherapy drugs, angiogenic agents and immunotherapy (platinum chemotherapy, topoisomerase inhibitors, anti-VEGF (anti-vascular endothelial growth factor), PI3K/AKT inhibitor, etc.) was used against solid tumors in clinical development [81].

The main target points, such as angiogenesis and the alteration of immune response also have a crucial role in diabetic retinopathy (DR) pathophysiology. Co-targeting pathways could offer new molecular therapeutic approaches for improved treatment and help moderate disease progression.

PARP-1 inhibitors alone have not been effective in treating DR because, while they can reduce inflammation and oxidative stress, they do not fully prevent the progression of the disease (no effect on vascular complications and neuroprotection). Recent studies explore novel receptor inhibitors and agonists, such as aldose reductase inhibitors [82,83,84], angiotensin-converting enzyme inhibitors [85,86] and peroxisome proliferator-activated receptor alpha agonists [87,88] (such as Fenofibrate and Pemafibrate [89]). Simultaneously, nanotechnology-based drug delivery systems address the solubility and penetration challenges. Their purpose is to overcome limitations associated with conventional treatments, enhance drug delivery to the retina and enable precise targeting [90,91,92]. Combining PARP-1 inhibitors with other therapies, such as VEGF, Rho-associated protein kinase inhibitors or neuroprotective peptides also offer a new option in DR treatment. The Pituitary adenylate cyclase-activating polypeptide (PACAP) and olaparib co-treatment of diabetic hypertensive rats was curative against structural and cellular changes in the retina. [93]. Besides PACAP, the beneficial effects of several neuropeptides were also confirmed in DR pathology. Other endogen substances, PEDF production increased after PARP inhibitor (PJ-34) treatment in endothelial cells under high glucose conditions [94]. These combinations target multiple pathways, potentially reducing inflammation, oxidative stress and neovascularization more effectively, leading to better clinical outcomes [93,95].

## 5. Conclusions

The combination of poly (ADP-ribose) polymerase-1 (PARP-1) inhibitors with other promising therapeutic agents could provide unique treatment solution against the pathophysiology of several retinal disorders. The concomitant targeting of signaling pathways with two or more drugs should require extensive caution, because dosage and potential side effects may ruin the benefits. In the future perspectives of diabetic retinopathy (DR) drug research, more attention should be given to the efficient combination of PARP-1 inhibitors with neuroprotective chemicals, such as neuropeptides. The emerging advantages of these combinations could offer new curative prospectives in the care of diabetic patients.
